# Comment on: Learning Curve From 450 Cases of Robot-Assisted Pancreaticoduodenectomy in a High-Volume Pancreatic Center

**DOI:** 10.1097/AS9.0000000000000137

**Published:** 2022-02-14

**Authors:** Fiona Hand, Tamara Gall, Long R Jiao

**Affiliations:** From the Department of Academic Surgery, The Royal Marsden Hospital, Imperial College London, London SW3 6JJ

## To the editor,

We read with interest the findings of Peng et al. recently published in the Annals of Surgery.^1^ Adoption of minimally invasive approaches to pancreaticoduodenectomy (PD) among the HPB community has been slow globally owing to its technical complexity. The utilisation of a robotic platform has the potential to address the technical difficulties encountered with laparoscopic PD; providing 3D vision with increased depth perception, increased range of motion due to wristed instruments, standardisation of surgery through the same instruments, and elimination of both instrument and camera tremor and variability. Indeed, the learning curve for basic surgical skills is significantly less with a robotic compared to the laparoscopic platform.^2^ In reviewing their database of 450 robotic-assisted PD the authors identify 250 cases as the point at which the learning curve is obtained. Interestingly, this coincided with the establishment of a standardised 10 step approach to all robotic PD at their centre.

Although we applaud the authors on a thorough study with a large patient cohort, a number of key points made in this article may prohibit other HPB centres from introducing robotic PD. The sample size cited by the authors needed to achieve the learning curve in robotic PD is substantial. Even for “high volume” PD centres solely utilising the robotic platform for all procedures, to complete the 250 cases deemed necessary for optimum operative performance would take at least 5 years. Furthermore, the patient cohort described is not readily comparable to that encountered in Western Europe or indeed the US with 95.8% ASA 2 or below and a mean BMI of 23.1. Similarly, the indication for surgery was malignant disease in just half of the patient cohort, 91% of whom had small T1 or T2 pancreatic ductal adenocarcinoma, which in the presence of a soft gland and a non obstructed small duct likely contributed to the high rate of postoperative pancreatic fistula documented by the authors. The patient cohort described in itself limits the applicability of robotic PD beyond the fittest patients, ironically when it is the more comorbid, higher BMI patient that tends to benefit most from minimally invasive approaches.^3^

Like the authors, our experience of robotic PD has demonstrated a clear association between the establishment of a standardised step-by-step operative approach and the attainment of proficiency. However, we instituted this standardised approach after the first 20 robotic PD, achieving a reduction in operative time from 470 to 308 mins (*P* < 0.001) without any increase in postoperative morbidity or reoperation rates. Before beginning robotic PD, our team had experience in more than 100 laparoscopic PD and robotic distal pancreatectomy, which other centres have attributed to a shorter learning curve.^4^ The authors of this present study note a 30% incidence of the postoperative pancreatic leak (either biochemical or established POPF) in the first 100 cases, which fell to 18% for cases 251–300. This improvement they attribute to the attainment of “visual feedback”, a surrogate for loss of haptics in robotic surgery which, according to the authors was acquired through their large sample size of robotic PD. The acquisition of “visual feedback” in performing robotic surgery is essential to enable judgement of tension when suturing or indeed performing anastomoses.^5^ Perhaps owing to our already established minimally invasive practice, visual feedback was established early and resulted in a postoperative pancreatic leak rate of 9.5% in our first 20 cases and 6.8% in subsequent cases (*P* = 0.72). Furthermore, in standardising our operative approach to robotic PD, we utilise a modified Blumgart technique for pancreaticojejunal anastomosis, in line with the Pittsburgh group,^6^ which may have a shorter learning curve that described in this study. Although the authors do not divulge the preexisting experience of the surgeons included in the analysis, this would prove an interesting point for the development of minimally invasive trials going forward.

The Miami International Evidence-Based Guidelines on Minimally Invasive pancreas resection has concluded that surgeon volume is associated with outcomes for minimally invasive PD. However, they concede the quality of the existing evidence is weak.^7^ To date no consensus exists on the volume needed to attain proficiency in robotic PD. Our experience mirrors the findings in this present study that the establishment of a standardised operative approach is key to achieving proficiency in robotic PD, optimising procedure time and minimising postoperative complications. While we congratulate the authors on what is a substantial volume of robotic PD, we feel that this publication should not deter the advancement of minimally invasive PD in high volume HPB centres where mentorship and the introduction of standardised PD training programmes has been shown to significantly shorten the learning curve.^8,9^

## References

[1] Shi Y Wang W Qiu W. Learning curve from 450 cases of robot-assisted pancreaticoduocectomy in a High-Volume Pancreatic Center: Optimization of Operative Procedure and a Retrospective Study. Ann Surg. 2021;274:e1277–e1283. 31651533 10.1097/SLA.0000000000003664

[2] Gall TMH Alrawashdeh W Soomro N. Shortening surgical training through robotics: randomized clinical trial of laparoscopic versus robotic surgical learning curves. BJS Open. 2020;4:1100 1108. 33052038 10.1002/bjs5.50353 PMC7709379

[3] Konstantinidis IT Lewis A Lee B. Minimally invasive distal pancreatectomy: greatest benefit for the frail. Surg Endosc. 2017;31:5234–5240. 28493165 10.1007/s00464-017-5593-y PMC5980234

[4] Zhang T Zhao ZM Gao YX. The learning curve for a surgeon in robot-assisted laparoscopic pancreaticoduodenectomy: a retrospective study in a high-volume pancreatic center. Surg Endosc. 2019;33:2927–2933. 30483970 10.1007/s00464-018-6595-0

[5] Reiley CE Akinbiyi T Burschka D. Effects of visual force feedback on robot-assisted surgical task performance. J Thorac Cardiovasc Surg. 2008;135:196–202. 18179942 10.1016/j.jtcvs.2007.08.043 PMC2674617

[6] Cai J Ramanathan R Zenati MS. Robotic pancreaticoduodenectomy is associated with decreased clinically relevant pancreatic fistulas: a propensity-matched analysis. J Gastrointest Surg. 2020;24:1111–1118. 31267434 10.1007/s11605-019-04274-1

[7] Asbun HJ Moekotte AL Vissers FL; International Study Group on Minimally Invasive Pancreas Surgery (I-MIPS). The Miami International evidence-based guidelines on minimally invasive pancreas resection. Ann Surg. 2020;271:1–14. 31567509 10.1097/SLA.0000000000003590

[8] Al Abbas AI Wang C Hamad AB. Mentorship and formal robotic proficiency skills curriculum improve subsequent generations’ learning curve for the robotic distal pancreatectomy. HPB (Oxford). 2021;23:1849–1855. 34059420 10.1016/j.hpb.2021.04.022

[9] Rice MK Hodges JC Bellon J. Association of mentorship and a formal robotic proficiency skills curriculum with subsequent generations’ learning curve and safety for robotic pancreaticoduodenectomy. JAMA Surg. 2020;155:607–615. 32432666 10.1001/jamasurg.2020.1040 PMC7240650

